# Calibration Approach for Gaseous Oxidized Mercury Based on Nonthermal Plasma Oxidation of Elemental Mercury

**DOI:** 10.1021/acs.analchem.2c00260

**Published:** 2022-06-01

**Authors:** Jan Gačnik, Igor Živković, Sergio Ribeiro Guevara, Jože Kotnik, Sabina Berisha, Sreekanth Vijayakumaran Nair, Andrea Jurov, Uroš Cvelbar, Milena Horvat

**Affiliations:** † Department of Environmental Sciences, Jožef Stefan Institute, Jamova Cesta 39, 1000 Ljubljana, Slovenia; ‡ Jožef Stefan International Postgraduate School, Jamova Cesta 39, 1000 Ljubljana, Slovenia; § Laboratorio de Análisis por Activación Neutrónica, Centro Atómico Bariloche, Av. Bustillo km 9.5, 8400 Bariloche, Argentina; ∥ Department of Gaseous Electronics, Jožef Stefan Institute, Jamova Cesta 39, 1000 Ljubljana, Slovenia

## Abstract

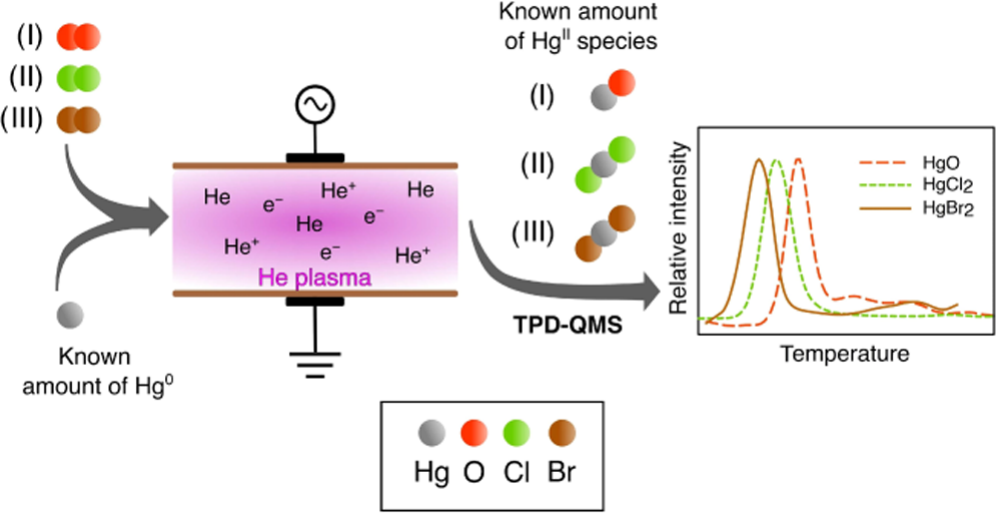

Atmospheric mercury measurements carried out in the recent decades have been a subject of bias largely due to insufficient consideration of metrological traceability and associated measurement uncertainty, which are ultimately needed for the demonstration of comparability of the measurement results. This is particularly challenging for gaseous Hg^II^ species, which are reactive and their ambient concentrations are very low, causing difficulties in proper sampling and calibration. Calibration for atmospheric Hg^II^ exists, but barriers to reliable calibration are most evident at ambient Hg^II^ concentration levels. We present a calibration of Hg^II^ species based on nonthermal plasma oxidation of Hg^0^ to Hg^II^. Hg^0^ was produced by quantitative reduction of Hg^II^ in aqueous solution by SnCl_2_ and aeration. The generated Hg^0^ in a stream of He and traces of reaction gas (O_2_, Cl_2_, or Br_2_) was then oxidized to different Hg^II^ species by nonthermal plasma. A highly sensitive ^197^Hg radiotracer was used to evaluate the oxidation efficiency. Nonthermal plasma oxidation efficiencies with corresponding expanded standard uncertainty values were 100.5 ± 4.7% (*k* = 2) for 100 pg of HgO, 96.8 ± 7.3% (*k* = 2) for 250 pg of HgCl_2_, and 77.3 ± 9.4% (*k* = 2) for 250 pg of HgBr_2_. The presence of HgO, HgCl_2_, and HgBr_2_ was confirmed by temperature-programmed desorption quadrupole mass spectrometry (TPD-QMS). This work demonstrates the potential for nonthermal plasma oxidation to generate reliable and repeatable amounts of Hg^II^ compounds for routine calibration of ambient air measurement instrumentation.

Atmospheric mercury is the largest pool of anthropogenic Hg.^1^ Oxidized mercury (Hg^II^) is present in the atmosphere either directly due to emissions or indirectly through the oxidation of elemental mercury. Hg^II^ can be methylated and bioaccumulated into the food chain after entering ecosystems via wet and dry deposition to aquatic and terrestrial environments.^2^ The degree of dry and wet deposition in global Hg assessments can only be estimated by knowing the chemistry and composition of atmospheric Hg^II^ species.^3^ Atmospheric Hg^II^ species are usually presented as operationally defined gaseous oxidized mercury (GOM), particulate-bound mercury (PBM), or reactive mercury (RM, GOM + PBM). Even though some Hg^II^ species, such as HgCl_2_ and HgBr_2_, have been identified in the atmosphere,^4,5^ the exact composition of atmospheric Hg^II^ remains unknown; this points to the need for improved atmospheric mercury speciation.^6^

The first problems associated with atmospheric mercury speciation were identified more than a decade ago. The most commonly used procedure for atmospheric mercury speciation uses preconcentration on KCl-coated denuders that are subject to biases. Biases originate from the low GOM collection efficiency of denuders in the presence of ozone and high humidity.^7−10^ Another analytical challenge is the correct calibration or lack thereof, as currently the calibration for GOM measurements is performed using Hg^0^ vapor. Moreover, Hg^0^ vapor concentration and its temperature dependence are described by an empirical equation that is not universally agreed upon.^11,12^ Additionally, instruments should be calibrated directly with gaseous Hg^II^ species instead of Hg^0^ to ensure a valid calibration.^13^ Currently, the available calibrations for oxidized mercury species are based on either permeation^14,15^ or liquid evaporation of Hg^II^ salts.^16,17^ Although permeation calibrators are promising, they are still in the development stage, showing inconsistent results at permeation rates relevant to ambient Hg^II^ concentrations.^18^ Liquid-evaporative calibrators perform well at flue gas Hg^II^ concentrations while having a biased low output at ambient Hg^II^ concentrations, mainly due to the adsorption and reactive nature of Hg^II^.^19^ Permeation and liquid-evaporative calibrators may also be subject to Hg^0^ impurities.^14^ No calibration is currently available for PBM measurements. Due to the aforementioned problematics of atmospheric mercury speciation, measurement results are still a subject of biases and thus cannot be reliably evaluated as equivalent.

Validation of the calibration and, in general, validation of the methodology for atmospheric mercury speciation can be effectively assessed utilizing labeled Hg species. Although the use of Hg stable isotopes is prevalent in the literature,^20−24^ radioactive isotopes offer certain advantages. The use of the ^197^Hg radiotracer (half-life 2.671 days) enables validation at ambient concentrations due to its high specific activity and absence of blanks and contamination issues (^197^Hg is not present in the nature).^19,25,26^

Nonthermal plasmas (NTP) differ from thermal and high-temperature plasmas in terms of the output energy conversion: in nonthermal plasmas, most of the energy is used to produce energetic electrons, while in thermal plasmas, the energy is also converted into heat.^27^ NTP can be generated under near-ambient conditions (room temperature and atmospheric pressure) by means of corona discharges, dielectric barrier discharges (DBD), atmospheric pressure plasma jet (APPJ), micro hollow cathode discharges (MHCD), and many more, all having their own distinctive properties and applications.^28^ To the best of the authors’ knowledge, Chen et al.^29^ were the first to employ NTP using DBD as a method for the oxidation of Hg^0^ in flue gases. The method was found to be cost-effective with good oxidation efficiency (up to 80%) in the simulated flue gas mixture.^29^ Oxidation reaction pathways,^30,31^ the influence of flue gas composition^32,33^ and the improvement of the Hg^0^ oxidation efficiency by CaCl_2_ treatment^34^ were all investigated for the oxidation of Hg^0^ in flue gases with NTP.

The use of DBD-NTP (hereinafter abbreviated as NTP) has so far been largely limited to the removal of Hg^0^ from flue gases. In this paper, we report the development of a novel calibration system for gaseous Hg^II^ species based on the oxidation of Hg^0^ to Hg^II^ by NTP. Using a highly selective and sensitive ^197^Hg radiotracer, the newly developed calibration method was validated for use at ambient Hg^II^ concentrations.

## 1 Experimental Section

The validation experiments were performed using a ^197^Hg radiotracer. The use of ^197^Hg is not an essential part of the calibration but a useful tool that has been used for validation. For real-time calibration, the use of “normal” or nonradioactive Hg, such as National Institute of Standards and Technology (NIST) standard reference material (SRM) 3133, is intended. Real-time calibration instructions are described in the Supporting Information, Section S1, in the standard operating procedure (SOP) format.

All chemicals and instruments that were utilized in the following experiments are listed in the Supporting Information, Section S2.

### 1.1 Production of ^197^Hg Radiotracer

^197^Hg radiotracer was used for the majority of performed experiments. Mercury enriched to 51.58% in ^196^Hg isotope (0.15% natural abundance) was used for irradiation to produce a ^197^Hg radiotracer. Enriched ^196^Hg was diluted in 2% HNO_3_ acid (v/v) solution and sealed into a quartz ampoule. By irradiating the ampoule with a high neutron flux (10^13^ cm^–2^ s^–1^) for 12 h in the central channel (CC) of the 250 kW TRIGA Mark II research reactor (Jožef Stefan Institute, Ljubljana, Slovenia), ^197^Hg (*t*_1/2_ = 2.671 d) was formed via a neutron capture reaction (n,γ).^26^ Similar reactions occur also for the formation of ^199m^Hg (*t*_1/2_ = 0.0296 d) from ^198^Hg, ^203^Hg (*t*_1/2_ = 46.594 d) from ^202^Hg, and ^205^Hg (*t*_1/2_ = 0.0036 d) from ^204^Hg. Nevertheless, ^199m^Hg, ^203^Hg, and ^205^Hg were never measured in our experiments due to their lower specific activity. Other Hg isotopes were unaffected by the neutron flux. After irradiation, the ^197^Hg^II^(aq) stock solution was diluted to 100 pg mL^–1^ which was used as the working solution for the experiments.

### 1.2 Determining ^197^Hg Using an HPGe Detector

The activity of ^197^Hg was determined using two approaches, depending on the Hg collection protocol. The activity of ^197^Hg collected on gold sorbent traps was measured by means of a coaxial-type HPGe detector, while in solutions, the activity was measured using a well-type HPGe detector. All activity measurements were relative to standards obtained from the irradiated solution in each experimental run. Peak area comparison of the sample and standard activity for the characteristic doublet peaks of γ-ray and X-ray emissions (67.0 + 68.8 and 77.3 + 78.1 keV) was performed using Genie 2000 Gamma analysis software. Oxidation and thermal reduction efficiencies were calculated as shown in the Supporting Information, Section S3.

Standards for the coaxial-type HPGe detector were obtained by a reduction of ^197^Hg^II^(aq) to ^197^Hg^0^(g), using a tin(II) chloride (SnCl_2_) solution (100 mL, 2% SnCl_2_ (w/v) and 0.5% HCl (v/v)). Produced ^197^Hg^0^ was purged for 10 min with N_2_ carrier gas (purity 4.7, flow rate of 1 L min^–1^) and captured by a downstream gold trap to obtain a measurement standard. Gold traps were prepared out of gold-coated Al_2_O_3_ (corundum) as described in previous work.^35^ To obtain standards for a well-type HPGe detector, triplicates of a Hg radiolabeled solution (8 mL, 2% HNO_3_ (v/v)) were transferred into glass vials and measured by a well-type HPGe detector.

The values discussed in the Section 2 were obtained by comparing the sample activities to the standard activities. To determine the equivalence between the ^197^Hg activity level and the Hg amount (including all Hg isotopes), the activity of stock ^197^Hg^II^(aq) solution was connected to its concentration by cold vapor atomic absorption spectroscopy (CV-AAS) measurement (calibration against NIST SRM 3133).^36^ The concentration of stock ^197^Hg^II^(aq) solution was 93.3 μg mL^–1^ of Hg. CV-AAS measurements were performed before and after irradiation to ensure the stability of Hg concentration in the stock solution.

### 1.3 Production of Hg^II^ Species by Nonthermal Plasma

A simplified scheme of the experimental design is shown in Figure 2a. In the first step, Hg^0^ was produced in a 250 mL impinger by the reduction of ^197^Hg^II^(aq) working solution using SnCl_2_ (as in Section 1.2.). From 1 to 2.5 mL of 100 pg mL^–1 197^Hg^II^(aq) working solution was used to produce 100–250 pg of Hg^0^. This amount was chosen as it is sufficiently low to imitate ambient Hg levels while still assuring low measurement uncertainty of the activity measurement.

**Figure 1:**
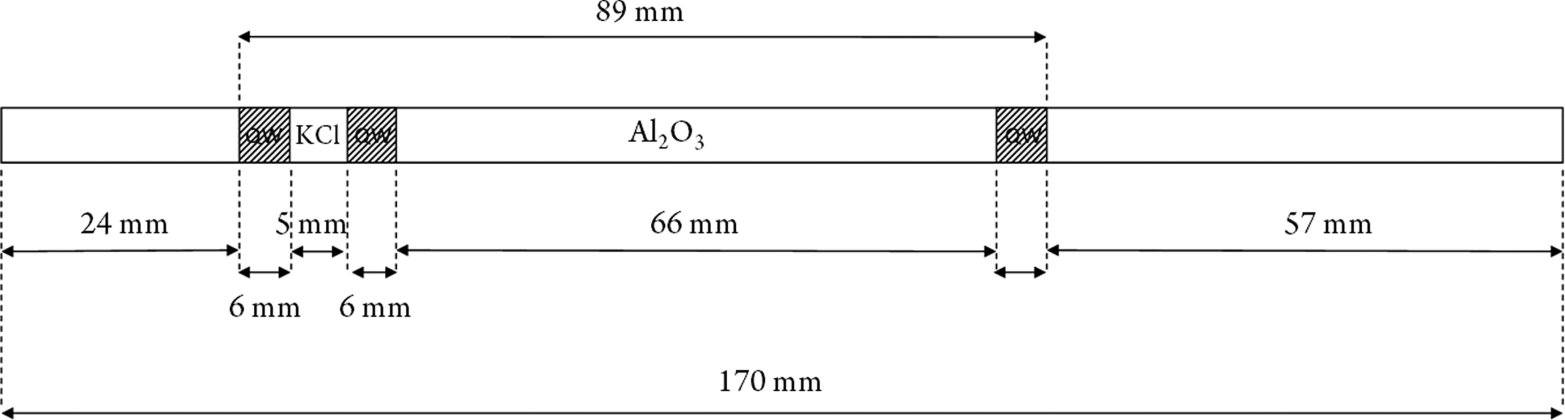
Design of the plasma trap, implemented for NTP oxidation of Hg^0^ to Hg^II^ (QW—quartz wool).

Produced Hg^0^ was aerated from the reaction solution for 10 min using nitrogen gas (1400 mL min^–1^), dried using a soda lime trap, and collected on the primary gold trap. The primary gold trap was then transferred to a separate setup that was used for the second step of NTP loading. In the second step, the primary gold trap was heated at 400 °C which released the trapped Hg^0^ into the stream of He gas (gas flow of 370 mL min^–1^). Downstream, Hg^0^ and He carrier gas were mixed with trace amounts of reaction gas (O_2_, Cl_2_, or Br_2_). O_2_ was obtained from a gas cylinder (purity 5.0), while Cl_2_ and Br_2_ were produced by electrolysis of 1 mol L^–1^ NaCl and KBr solution, respectively. More details regarding the electrolytic production of Cl_2_ and Br_2_ are available in the Supporting Information, Section S4. Reaction gas was mixed with He and Hg^0^ downstream of the primary gold trap. The resulting gas mixture consisted of 99.2% of He and 0.8% of O_2_, while for Cl_2_ and Br_2_, their relative flows were about 0.5% due to the partial solubility of Br_2_ and Cl_2_ in the KBr or NaCl electrolytic solution. In the mixture of He and the reaction gas, Hg^0^ was then oxidized to Hg^II^ by NTP. Hg^II^ was captured on the spot by the KCl crystals, while traces of unoxidized fraction of Hg^0^ (breakthrough) were collected on a gold trap. The oxidation efficiency was determined according to the analytical protocol described in Section 1.2. The most important parameter for the NTP oxidation system was the design of the dielectric quartz tube used as “plasma trap” shown in Figure 1. The use of Al_2_O_3_ catalyst is explained in Section 2.1.

NTP was ignited using a high-voltage high-frequency power generator by applying voltage to a pair of copper electrodes. Electrodes were made of 1 mm thick copper plates, which were cut into 10 mm wide strips and bent around the quartz tube. Such copper strips were separated 5 mm from each other when placed on the plasma trap. By controlling the input power (and indirectly voltage and current) of the generator, we controlled the NTP parameters. The obtained optimal parameters of the NTP generator were: average power applied to the electrodes of 180 μW, radiofrequency of 20 kHz, effective voltage of 345 V, effective current of 7.0 mA, and the phase angle between voltage and current of −101°. The presented values were measured for the gas combination of He and O_2_, but they were similar for other gas mixtures.

### 1.4 Thermal Reduction of Hg^II^ to Hg^0^ on Sorbent Traps

**Figure 2:**
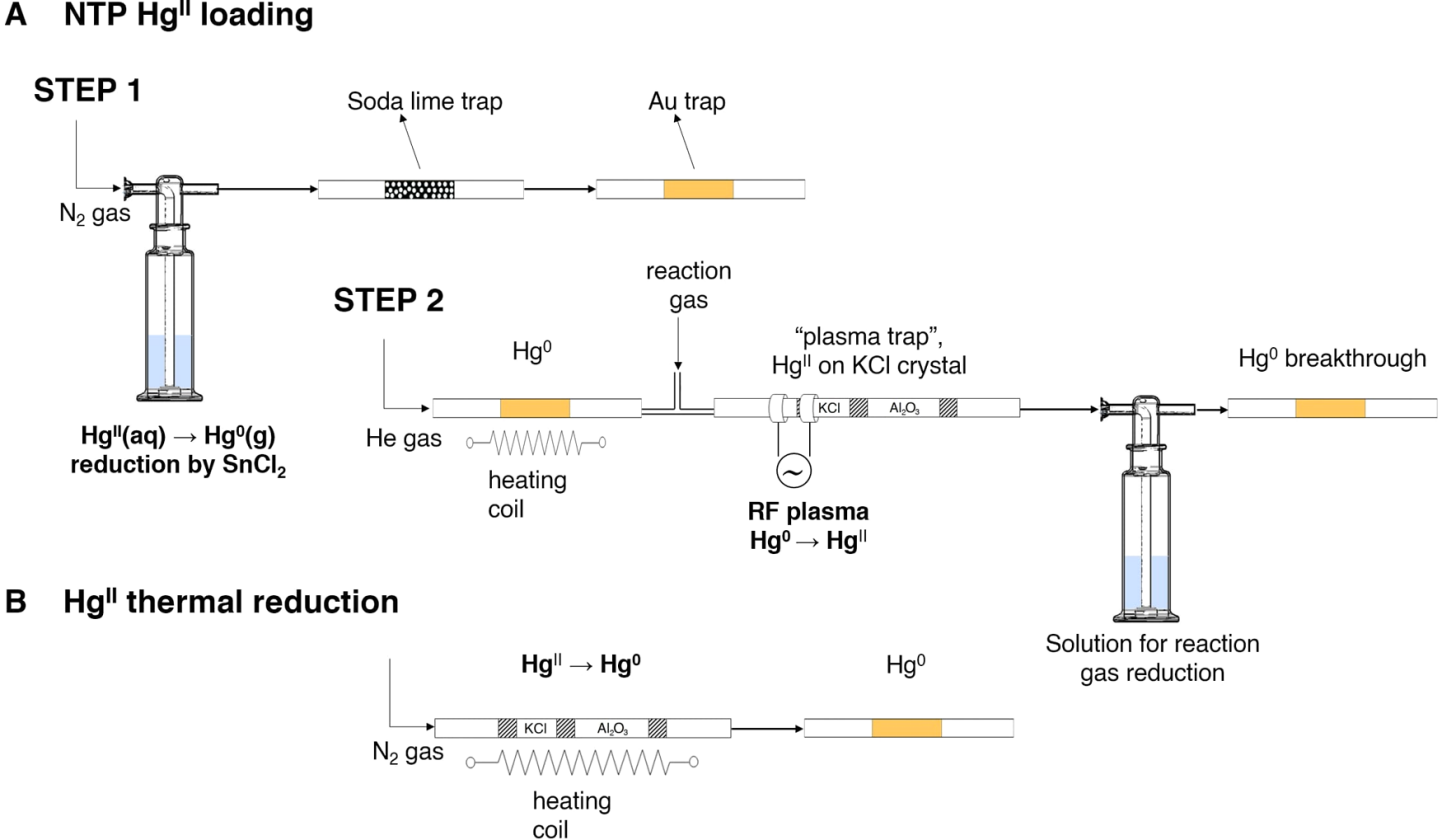
(A) Experimental setup for nonthermal plasma (NTP) loading of Hg^II^ species: in step 1, the gold trap is loaded with Hg^0^ by purge and trap. In step 2, the loaded Hg^0^ is desorbed from the gold trap and oxidized to Hg^II^ by NTP in a stream of helium and reaction gas mixture. (B) Experimental setup for Hg^II^ to Hg^0^ thermal reduction and reduction efficiency studies: Hg^II^ loaded on the KCl is reduced to Hg^0^ in the stream of N_2_ by Al_2_O_3_ catalyst-assisted thermal reduction. The reduced Hg^0^ is captured by the gold trap.

Traps with KCl crystal and tested catalyst material (or “plasma traps”) were also used for the thermal reduction experiments to assure quantitative reduction. ^197^Hg^II^ was loaded onto the KCl crystal part by spiking 10 μL of ^197^Hg^II^(aq) in a 2% HNO_3_ solution. The actual amount of Hg^II^ depended on the extent to which the radiotracer had already decayed and ranged between 100 and 500 pg. The design shown in Figure 2b was used to study the Hg^II^ thermal reduction efficiency.

Both the KCl crystal and the catalyst part were heated (ramped) from room temperature to 600 °C in 20 s. The trap was vented using N_2_ carrier gas (flow rate of 370 mL min^–1^) for 60 s after the end of heating to ensure complete downstream transport of ^197^Hg^0^ to a gold trap. ^197^Hg^0^ on the gold trap was measured using a γ coaxial detector.

Unconverted ^197^Hg^II^ on the KCl trap was washed from the quartz tube and leached from the trap using a previously determined optimal washing solution (20 mL of 10% HNO_3_ (v/v) + 5% HCl (v/v) solution). The washing solution (8 mL) was taken into the measurement vial, and the activity of the solution was measured with a γ well detector.

### 1.5 Analysis of Hg^II^ Species Using Temperature-Programmed Desorption

Indirect analysis of Hg^II^ species was performed on the basis of temperature-programmed desorption (TPD) using a quadrupole mass spectrometer (QMS) operating under a high vacuum. The experimental design of the TPD-QMS analysis of Hg was based on previous work.^37^ Samples (<5 mg) loaded into a heating cell were heated to 750 °C at a rate of 10 °C min^–1^. Heated desorbed atoms or molecules were then ionized at 70 eV using a cross-beam ion source followed by QMS separation based on mass-to-charge (*m*/*z*) ratio and detected using a secondary electron multiplier detector. Two different stable Hg isotopes were measured simultaneously, ^200^Hg and ^202^Hg. Data for ^202^Hg were used to prepare TPD spectra for all analyzed Hg^II^ species. Hg^II^ species were determined indirectly because the direct determination of ^202^HgX_n_ (X=O, Cl, or Br) is not possible; therefore, only ^202^Hg was measured directly.

Using TPD-QMS, we measured two kinds of samples: Al_2_O_3_ (corundum) with Hg^II^ species loaded by NTP and Al_2_O_3_ mixed with Hg^II^ species standards. Hg^II^ species were loaded on Al_2_O_3_ using NTP as described in Section 1.3, with the only difference being that NIST 3133 was used instead of the ^197^Hg radiotracer. Hg^II^ species standards were prepared using the so-called “wet preparation” method. For the wet preparation method, we used 100 mL of 1 mg mL^–1^ HgO, HgCl_2_, and HgBr_2_ solutions and added 0.5 g of Al_2_O_3_. The resulting solution containing insoluble Al_2_O_3_ was then stirred for 30 min, centrifuged, and air-dried. Al_2_O_3_ prepared in this way served as a Hg^II^ species standard for TPD-QMS.

## 2 Results and Discussion

Initial results showed that the efficiency of NTP oxidation of Hg^0^ to Hg^II^ could not be reliably estimated if the produced Hg^II^ was incompletely converted to Hg^0^ by thermal reduction (Figure 2b). Therefore, we first tested various catalysts that could promote higher efficiency of thermal reduction of Hg^II^ to Hg^0^. After selecting the most suitable catalyst, we examined the production of Hg^II^ by the NTP oxidation of Hg^0^. Finally, we performed measurements using TPD-QMS with an attempt to confirm the presence of different Hg^II^ species that were produced by NTP oxidation. The order of presenting the results and discussion is therefore in the same sequence as described in the above paragraph.

### 2.1 Thermal Reduction of Hg^II^ to Hg^0^ on Sorbent Traps

We studied the efficiency of thermal reduction for Hg^II^ loaded by spiking the plasma trap. Optimization of the thermal reduction was crucial to ensure reliable estimation of the NTP oxidation efficiency, which is discussed in Section 2.2. First results showed low reduction efficiencies; therefore, a set of different catalysts were used to promote the Hg^II^ thermal reduction. The catalysts were placed in the section of the “plasma trap” marked as “Al_2_O_3_” in Figure 1. Used catalysts were: Au-coated silica sand, densely packed platinum (Pt) wire, densely packed quartz wool, and Al_2_O_3_ (corundum, 0.60–0.85 mm grain size). The results of the experiments are shown in Table 1.

**Table 1:** Hg^II^ to Hg^0^ Thermal Reduction, Hg^II^ Loaded by Spiking^a^

catalyst used	Hg^0^ [%]	unconverted Hg^II^ [%]	mass balance [%]
none	88 (26)	25.6 (43)	113 (22)
Au-coated silica	38 (3)	61 (5)	99 (2)
Pt wire	39 (28)	49 (32)	88 (5)
quartz wool	86 (19)	15 (12)	101 (8)
Al_2_O_3_	101 (3)	<0.1	101 (3)

a Values are shown as averages of multiple replicates (replicates shown in Supporting Information, Section S5) with the repeatability standard deviation notation in the brackets.

When thermal reduction was not quantitative, Hg^II^ species were not completely reduced but partially desorbed from the plasma trap and deposited on the cooler parts of the tubing prior to reaching the gold trap. Initially, Pt wire as a catalyst showed promising results, but it was evident that with its reuse, the reduction efficiency decreased considerably between runs (1.92, 59.4, 64.8, and 71.5% of unconverted Hg^II^, listed in consecutive runs: see Supporting Information, Section S5). The decrease in efficiency was attributed to the passivation of Pt. Quartz wool performed better than Pt wire, but Al_2_O_3_ was selected as the best catalyst, having the highest and most repeatable reduction efficiency (Table 1). Due to the knowledge gained from Hg^II^ reduction experiments, only the Al_2_O_3_ catalyst was implemented into the final NTP design.

### 2.2 Production of Hg^II^ Species by Nonthermal Plasma

The method of producing gaseous Hg^II^ species by NTP utilizes the oxidation of Hg^0^ in plasma with oxidative gases and He carrier gas. The combination of plasma and oxidative gases seems to be effective in the quantitative production of gaseous Hg^II^ species under ambient air concentrations. The predicted Hg^II^ species that were produced were HgO using O_2_ as a reaction gas, HgCl_2_ using Cl_2_ as a reaction gas, and HgBr_2_ using Br_2_ as a reaction gas. Whether those exact species were actually produced is discussed in Section 2.3. The validity of the calibration for Hg^II^ species by NTP oxidation was tested by evaluating the oxidation efficiency for each investigated oxidation reaction. Four to five replicate measurements were performed for each species to assess the oxidation efficiency (all replicates shown in the Supporting Information, Section S5). The corresponding standard uncertainty of the developed calibration was estimated according to the GUM and Eurachem guidelines.^38,39^ Standard measurement uncertainties were assessed from all experimental data; repeatability contributed the most to the combined standard uncertainty for all three Hg^II^ species (relative contribution of 65, 81, and 94% for HgO, HgCl_2_, and HgBr_2_, respectively). The uncertainty contribution due to ^197^Hg activity measurement was substantially lower, indicating that the use of ^197^Hg is justified for the intended use. However, for the proper traceability to NIST 3133 (and as a consequence traceability to System of Units), the effects of blanks will be needed to be taken into account, as they will likely contribute significantly to the analytical signal and measurement uncertainty. This is especially true at very low (ambient) Hg^II^ concentrations. The complete standard uncertainty estimation procedure is given in the Supporting Information, Section S6.

Resulting oxidation efficiencies with corresponding expanded standard uncertainty values were 100.5% ± 4.7% (*k* = 2) for 100 pg of HgO, 96.8% ± 7.3% (*k* = 2) for 250 pg of HgCl_2_, and 77.3% ± 9.4% (*k* = 2) for 250 pg of HgBr_2_. The provided masses refer to the amount of Hg^0^ used for oxidation to Hg^II^. Similar ambient-level masses of Hg^II^ as used in our work are usually sampled for atmospheric Hg speciation. For example, 125 min sample preconcentration using 8 L min^–1^ airflow of ambient air with 100 pg m^–3^ Hg^II^ concentration gives 100 pg of preconcentrated Hg^II^.

While the oxidation efficiency values for HgO and HgCl_2_ indicated almost quantitative oxidation, the oxidation efficiencies of HgBr_2_ were considerably lower. There are two contrasting effects that might influence this observation. First, the low recoveries of HgBr_2_ might be due to the electrolytic production of Br_2_ gas. When Br_2_ is produced from the electrolytic solution, it dissolves immediately, so a large amount of Br_2_ must be produced to achieve a sufficiently high vapor pressure of aqueous Br_2_. Otherwise, the small amount of Br_2_ in the carrier gas seemed insufficient for the complete oxidization of Hg^0^ in the NTP. Nevertheless, Br_2_ and Cl_2_ are highly reactive gases that can oxidize Hg^0^ even prior to the NTP part of the setup, resulting in losses due to adsorption of Hg^II^ on walls prior to the NTP section. To reduce losses, the distance between T-split (mixing of Cl_2_(g)/Br_2_(g) with Hg^0^(g)) and NTP (Figure 2) had to be minimized. Of the two described effects (insufficient amount of Br_2_ in the carrier gas and premature oxidation), we cannot assess which effect is more prevalent.

Due to the aforementioned properties of Cl_2_ and Br_2_, the highest recoveries and the lowest standard uncertainties were achieved using O_2_ as a reaction gas, as it introduces the least complexity into the experimental setup due to its relative inertness and availability. Repeatability could be improved in the future by automating the presented NTP calibration system.

Comparison of the NTP calibration system with other available Hg^II^ calibration systems is difficult due to (i) scarce data on the system accuracy and precision, (ii) data usually available only for high Hg^II^ concentrations (>1 μg m^–3^ of Hg), and (iii) lack of metrological traceability for the accuracy and precision data. Liquid evaporative calibrators developed by HovaCal, VTT & Optoseven, and Tekran (model 3315) are designed for use with high Hg^II^ concentrations. In our previous work, we evaluated the accuracy of a liquid-evaporative calibrator at near-ambient Hg^II^ concentration levels. The main conclusion was that while at the μg m^–3^ level, the error ranged from 19 to 4% (depending on the duration of operation time), at the ng m^–3^ level, the error increased to as much as 63%. Additionally, the precision was unsatisfactory due to the time dependence of the calibrator output.^19^ Therefore, the only calibration systems that can be compared to the NTP calibration system are those that were evaluated at ambient Hg^II^ concentration levels. As such, Hg^II^ permeation tubes are currently the only calibration system suitable for comparison. The permeation rates, their precision, and accuracy have been determined, but the data depended on measurement systems that can be subject to biases and are not traceable.^14^ The design and dimensions of each individual permeation tube affect the permeation rate; consequently, each unit has to be assessed individually. Lyman et al.^18^ attempted to establish the traceability of permeation tubes to the mass (and consequently to SI units), but low mass losses and weighing difficulties prevented reliable results.^18^ High permeation rates (up to 30 pg s^–1^) based on the gravimetric method matched the determined Hg concentrations to within 25%, but the agreement was worse for lower permeation rates.^18^ The NTP approach for Hg^II^ calibration is accurate (no observed bias when calibration uncertainty is considered) for HgO and HgCl_2_, while future optimization will be needed for HgBr_2_ calibration accuracy and precision. Although traceability to SI units was not demonstrated within this paper, future work is planned on achieving SI traceability via NIST SRM 3133.

### 2.3 TPD-QMS Analysis of Produced Hg^II^ Species

**Figure 3:**
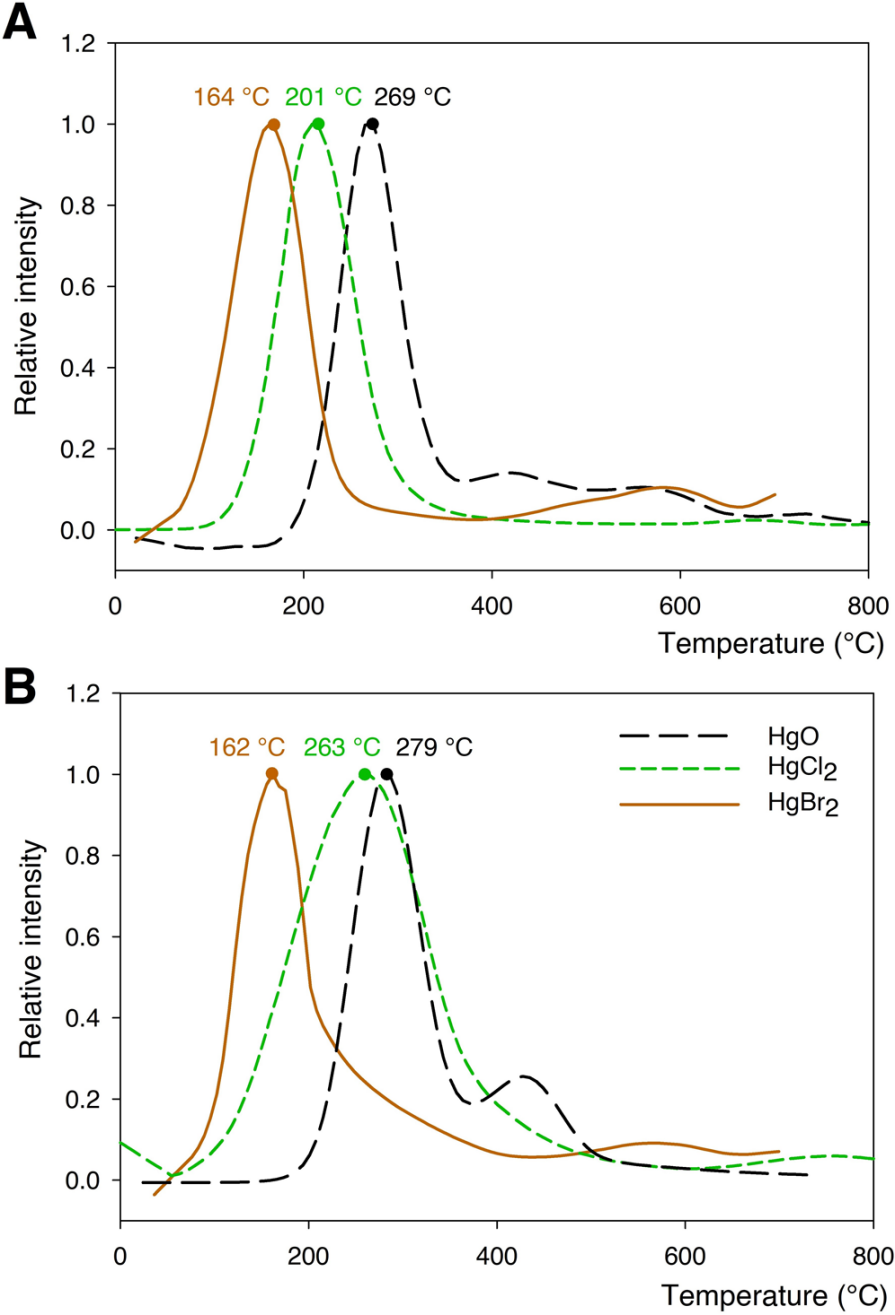
(A) Temperature-programmed desorption for three Hg^II^ species loaded on sorbent traps by NTP oxidation. (B) Results of the temperature-programmed desorption for Hg^II^ species standards. The temperatures indicated above the peaks are the temperatures of the highest signal intensity for each respective peak.

In addition to the evaluation of NTP oxidation efficiency, we attempted to confirm the presence of each species on the plasma trap by indirect TPD-QMS measurement. The results of TPD-QMS measurements are shown in Figure 3. Figure 3a shows the temperature-programmed desorption of Hg^II^ species obtained by NTP oxidation, while Figure 3b shows the results for Hg^II^ species standards.

TPD-QMS peaks for Hg^II^ species loaded by NTP oxidation and Hg^II^ species standards were compared to identify which species were produced. To simplify the discussion, we indexed the temperatures of the highest signal intensity as follows: *T*_HgO_, *T*_HgCl_2__, and *T*_HgBr_2__ notations for HgO, HgCl_2_, and HgBr_2_, respectively, and “NTP” or “STD” notations for NTP-loaded Hg^II^ species or for Hg^II^ species standards. *T*_HgO, NTP_ (269 °C) and *T*_HgO, STD_ (279 °C) are similar as well as *T*_HgBr_2_, NTP_ (164 °C) and *T*_HgBr_2_, STD_ (162 °C). *T*_HgCl_2_, NTP_ (201 °C) and *T*_HgCl_2_, STD_ (263 °C) are different (Figure 3), which can be explained by the wider peaks obtained for Hg^II^ species standards. The wider peaks could originate from the wet deposition method of preparing Hg^II^ species standards (Hg^II^ aqueous phase chemistry), which is not entirely equivalent to the NTP-loaded Hg^II^ species (Hg^II^ gas-phase chemistry). Considerable hydrolysis of HgCl_2_ in water and species transformation are possible, but those of higher halogenides (bromide and iodide) are less likely due to greater stability of these halogenides (several orders of magnitude lower solubility product constant *K*_sp_).^40,41^ Second, we compared the values obtained for NTP-loaded Hg^II^ species to the values from the literature. Literature values for temperature desorption of Hg^II^ species differ, mainly due to differences in the matrices to which Hg^II^ is bound. For HgO, the literature values for desorption temperature range from 240 to 310 °C for yellow HgO and from 550 to 600 °C for red HgO.^37,42^ The temperature desorption diagram for NTP-loaded HgO agrees well with the literature data for yellow HgO, which serves as additional confirmation that the Hg^II^ produced was really HgO. For matrix-bound HgCl_2_, the desorption temperature in the literature ranges between 190 and 250 °C,^37^ which is similar to our results for NTP-loaded HgCl_2_. The presence of Hg_2_Cl_2_ cannot be completely ruled out due to the fact that Hg_2_Cl_2_ and HgCl_2_ have very similar desorption temperatures.^43^ Nevertheless, desorption of Hg_2_Cl_2_ usually results in a doublet peak,^44^ which is not seen in our results. For HgBr_2_, there are not many data available for matrix-bound HgBr_2_ but mostly for pure HgBr_2_.^43^ HgBr_2_ is desorbed at lower temperatures than HgCl_2_ and HgO, which is in agreement with our observations.^43^ The limitation of our comparison to the literature values and to measured standards is that there is still a possibility that a similar but not yet tested Hg^II^ compound could have a similar temperature desorption range and therefore overlap with our presumably NTP-produced Hg^II^ species. Therefore, we can confirm the presence of presumed species with a high degree of confidence but not with absolute certainty.

## 3 Conclusions

Calibration using NTP oxidation of Hg^0^ to Hg^II^ has proven to be a suitable way to achieve quantitative production of two gaseous Hg^II^ species: HgO and HgCl_2_. They were produced with a degree of measurement uncertainty that we consider appropriate for ambient concentration calibration, as ambient Hg^II^ measurements are generally accompanied with higher uncertainty contributions (originating mostly from sampling). Quantitative production of HgBr_2_ was limited by the high gaseous reactivity or aqueous solubility of Br_2_ reaction gas. The presence of each produced Hg^II^ species was indirectly confirmed. Future research will focus on the use of NTP not only for sorbent traps but also for the calibration of denuders and the calibration of field measurements. Automation of the presented calibration could result in lower standard uncertainty due to improved repeatability.

## Supporting Information Available

Standard operating procedure (SOP) for the developed calibration method (Section S1); list of chemicals and instruments used in validation work (Tables S1 and S2); calculation of the decay time-corrected peak areas for samples and standards (Equations 1 and 2); calculation of the oxidation or thermal reduction efficiency (Equation 3); electrolytic production of reaction gases Cl_2_ and Br_2_ (Figure S1); all replicates for the Hg^II^ thermal reduction (Table S3) and production of Hg^II^ species by NTP (Table S4); calculation of the combined standard uncertainty (Equations 4 to 18); and a calculation example for HgCl_2_ species (Table S5) (PDF)
