# Supplementary material for: Calibration Approach for Gaseous Oxidized Mercury Based on Nonthermal Plasma Oxidation of Elemental Mercury
Source: Anal Chem. 2022 Jun 1;94(23):8234–40. doi: 10.1021/acs.analchem.2c00260 (PMC9201811; doi:10.1021/acs.analchem.2c00260)
Supplement: Supplementary file 1 — ac2c00260_si_001.pdf [file ac2c00260_si_001.pdf]

Supporting information

Calibration Approach for Gaseous Oxidized Mercury Based on Nonthermal Plasma Oxidation of Elemental Mercury

Jan Gačnik[1,2,‡], Igor Živković[1,‡], Sergio Ribeiro Guevara[3], Jože Kotnik[1,2], Sabina Berisha[2], Sreekanth Vijayakumaran Nair[1,2], Andrea Jurov[2,4], Uroš Cvelbar[2,4] and Milena Horvat[1,2,*]

[1]Department of Environmental Sciences, Jožef Stefan Institute, Jamova Cesta 39, 1000 Ljubljana, Slovenia

[2]Jožef Stefan International Postgraduate School, Jamova Cesta 39, 1000 Ljubljana, Slovenia

[3]Laboratorio de Análisis por Activación Neutrónica, Centro Atómico Bariloche, Av. Bustillo km 9.5, 8400 Bariloche, Argentina

[4]Department of Gaseous Electronics, Jožef Stefan Institute, Jamova Cesta 39, 1000 Ljubljana, Slovenia

[‡]J. G. and I. Ž. contributed equally to this work.

*E-mail: milena.horvat@ijs.si

Table of Contents

S1 - Standard operating procedure (SOP) for gaseous oxidized mercury species calibration by non-thermal plasma (NTP) oxidation of elemental mercury **S-1**

S2 - Chemicals and instruments used in the validation work **S-6**

Table S1. Chemicals used in the validation work S-6

Table S2. Instruments used in the validation work S-6

S3 - Calculation of the decay time-corrected peak areas for samples and standards and the calculation of oxidation or thermal reduction efficiency **S-7**

Equations (1) to (3) S-7

S4 - Electrolytic production of reaction gases Cl_2 and Br_2 .... **S-8**

Figure 1. Experimental setup for electrolytic production of reaction gases Cl_2 and Br_2 .... S-8

S5 - All replicates for the thermal reduction efficiency and NTP oxidation efficiency calculation **S-9**

Table S3. Hg^{II} to Hg^{0} thermal conversion, Hg^{II} loaded by spike, all replicates S-9

Table S4. Production of Hg^{II} species by NTP, all replicates S-9

S6 - Calculation of the combined standard uncertainty for the proposed Hg^{II} species calibration **S-10**

Equations (4) to (18) S-10, S-11

Table S5. Example: calculation of the combined standard uncertainty of $HgCl_2$ species calibration S-12

References **S-13**

S1 - Standard operating procedure (SOP) for gaseous oxidized mercury species calibration by non-thermal plasma (NTP) oxidation of elemental mercury

1. *Foreword and scope of the method*

The calibration method is based on generation of a known amount of gaseous elemental mercury (Hg^0), its quantitative oxidation to gaseous oxidized mercury species (Hg^{II}) by NTP in the presence of reaction gas, Hg^{II} thermal reduction back to Hg^0, and its determination by cold vapor atomic fluorescence spectrometry (CV-AFS). The method is designed to be suitable for calibration of any commercially available atmospheric mercury speciation unit. In the presented SOP, the medium used for Hg^{II} collection is a sorbent trap (in the following sections named "plasma trap"; see Figure 1 of the article's main body for more information). Instead of a sorbent trap, commonly used atmospheric Hg speciation methods often utilize denuders or sorbent membranes. Although these Hg^{II} collection methods can be used for calibration instead of sorbent traps, their compatibility with our calibration was not validated in our work. The instructions provided in this SOP are fit-for-purpose regardless of the method used for Hg^{II} collection and regardless of the method used for mercury detection.

The intended amounts of Hg^{II} species produced using this method for calibration of ambient concentration levels of Hg^{II} are in the order of 50-100 pg. Therefore, ultra-trace analysis protocols have to be applied to prevent contaminations: keeping the laboratory clean, providing appropriate ventilation, and adequately washing glassware, tools, and containers.

2. *Safety precautions*

Follow universal precautions. Wear gloves, a lab coat, and safety glasses whilst handling chemicals. For handling the non-thermal plasma and its power supply unit, the safety rules for working with electrical equipment should be followed: turn off the power supply of the equipment before inspecting it; use only tools/equipment with non-conducting handles; avoid contacting the setup with wet hands and wet materials; do not store highly flammable chemicals near the electrical equipment; call expert electrician if the power supply or instrument keeps causing burn-out of fuses.

3. *Chemicals, materials, and equipment*

3.1 Chemicals and materials

- 65% nitric acid (HNO_3, Suprapur, Merck, Germany)
- 30% hydrochloric acid (HCl, Suprapur, Merck, Germany)
- Potassium permanganate ($KMnO_4$, analytical grade, Merck, Germany)
- Potassium chloride (KCl, Suprapur, Merck, Germany)
- Potassium hydroxide (KOH, analytical grade, Merck, Germany)
- Sodium thiosulfate ($Na_2S_2O_3$, analytical grade, Merck, Germany)
- Potassium bromate ($KBrO_3$, Suprapur, Merck)
- Potassium bromide (KBr, Suprapur, Merck)

- Tin(II) chloride dihydrate ($SnCl_2 \cdot 2H_2O$, analytical grade, Merck, Germany)
- Hydroxylammonium chloride ($NH_2OH \cdot HCl$, analytical grade, Merck, Germany)
- Gold(III) chloride hydrate ($HAuCl_4{\cdot}xH_2O$, 99.995% trace metal basis, Merck, Germany)
- NIST SRM 3133: Mercury (Hg) Standard Solution (NIST, USA)
- Aluminum oxide, corundum (Al_2O_3, 0.60–0.85 mm grain size)
- Type I purified water (resistivity 18.2 MΩ cm)
- Quartz wool (Supelco, USA)

3.2 Equipment

- High-voltage high-frequency power generator (Information Unlimited, model Amazing PMV500, or similar)
- Cold vapor atomic fluorescence spectrometer (CV-AFS)

3.3 Preparation of reagents and materials

10% w/v $SnCl_2$ in 10% v/v HCl:

- Weigh accurately 10 g of $SnCl_2$ into a clean glass beaker using a plastic spatula (beaker and spatula are used only for $SnCl_2$).
- Add 10 mL of concentrated HCl directly to the $SnCl_2$ and transfer to a 100-mL volumetric flask. Mix and wait for complete dissolution of $SnCl_2$.
- Add Milli-Q water to the mark (100 mL).
- Purge the $SnCl_2$ solution with Hg-free nitrogen gas for two hours in order to obtain a mercury-free solution.

16% w/v $NH_2OH \cdot HCl$ solution:

- Weight 16 g of $NH_2OH \cdot HCl$ in a suitable glass bottle, add Milli-Q water to a 100-mL mark, and mix well.
- Add 20 μL of $SnCl_2$ solution and purge for 2 hours with Hg-free nitrogen gas.

10% w/v KOH:

- Dissolve 10 g of KOH in Milli-Q water to the final volume of 100 mL.
- Purge the KOH solution with nitrogen for two hours in order to obtain a mercury-free solution.

10% w/v $Na_2S_2O_3$:

- Dissolve 10 g of $Na_2S_2O_3$ in Milli-Q water to the final volume of 100 mL.
- Purge the $Na_2S_2O_3$ solution with nitrogen for two hours in order to obtain a mercury-free solution.

0.5% w/v $KMnO_4$ solution:

- Dissolve 0.5 g of $KMnO_4$ in Milli-Q water to the final volume of 100 mL.

Bromine monochloride (BrCl) oxidizing solution:

- Weigh 15 g of $KBrO_3$ and 11 g of KBr into a clean 1-liter glass bottle.
- Add 200 mL of Milli-Q water.
- Add 800 mL of concentrated HCI. The dilution has to be carried out in a well-ventilated fume hood to prevent exposure to toxic fumes released during dissolution of $KBrO_3$.
- Keep the bottle wrapped with aluminum foil.
- The prepared solution can be kept for an unlimited time if stored in darkness at 5 °C in a tightly closed Teflon or glass bottle.

Gold-coated corundum and gold trap preparation:

- Gold-coated corundum: Dissolve 1 g of $HAuCl_4 \cdot xH_2O$ in 10 mL of Milli-Q water and add 10 g of Al_2O_3. Evaporate the solution in an automatic rotary evaporator under reduced atmospheric pressure and then heat the remaining material at 500 °C for 4 h in an argon atmosphere.
- Gold trap preparation: Insert a small piece of quartz wool at the end of the longer part of the column. Then insert about 2 cm of gold-coated corundum. It is recommended to weigh the gold-coated corundum in order to obtain a better reproducibility between the traps. Insert a larger piece of quartz wool. Precondition the new trap by heating it at least 4 times before use.

Plasma trap preparation: shown in the main body of the article, in Figure 1.

Drying columns preparation: similar as for gold trap preparation, except that the gold-coated corundum is replaced with soda lime.

3.4 Mercury standard solutions (SRM NIST 3133)

Standard stock solution, 99.54 μg g^{-1} of Hg in 5% HNO_3: Important note: record the mass after each volume addition or subtraction step, otherwise the Hg mass concentration cannot be evaluated correctly. Fill a weighed 100-mL glass flask with 5% HNO_3 solution exactly to mark of 100 mL and remove 1 mL of the solution. Gently shake an ampoule of Standard Reference Material 3133, Mercury (Hg) Standard Solution for 2 minutes and open it according to the certificate. Take 1 mL of SRM solution with a pipette directly from the ampoule to the prepared glass flask containing acid solution. By doing so, a solution with an approximate Hg concentration of 99.54 μg g^{-1} is obtained (the exact value depends on the mass weighing). Cap the flask, shake it well and leave it on room temperature for 1 hour before further dilutions.

Intermediate and working standard solutions, 0.9954 μg g^{-1} of Hg in 5% HNO_3 and 0.9954 ng g^{-1} of Hg in 5% HCl: Prepare an intermediate Hg standard solution with the concentration of 0.9954 μg g^{-1} by 100-fold dilution of standard stock solution in the same manner as for standard stock solution. At the end, prepare a working Hg standard solution with the concentration of 0.9954 ng g^{-1} by 1000-fold dilution of the intermediate standard. The last dilution is made with 5% HCl solution instead of a 5% HNO_3 solution. Calculate the exact concentrations of the Hg standard solutions by taking into an account the exact mass of pipetted aliquots.

3.5 Cleaning glassware

Prior to use, thoroughly wash all laboratory glassware following the procedure:

- Allow the glass vessels to soak overnight in 2% Micro-90 detergent solution.
- Rinse the vessels thoroughly, first with tap water, and then with Milli-Q water.
- Rinse with 0.5% $KMnO_4$ solution.
- Rinse with water until the color of the $KMnO_4$ solution is no longer visible.
- Rinse with 2 mL of 16% $NH_2OH \cdot HCl$ solution.
- Rinse three times with Milli-Q water.
- Fill the vessels with 1% HCl solution and store in mercury-free storage facilities.
- Vessels should be emptied just before use for sample processing and allowed to dry at 60 °C.

4. *NTP calibration protocol*

As previously stated, the calibration method is comprised of three main steps: i) Hg^0 generation, ii) NTP oxidation of Hg^0 to Hg^{II} species in the presence of a reaction gas, and iii) thermal reduction of Hg^{II} species to Hg^0 with subsequent CV-AFS analysis. The second step has three different setup variations depending on the used reaction gas (O_2, Cl_2 or Br_2).

4.1 Hg^0 generation

Similar setup is used as in Figure 2a of the manuscript's main body.

- Provide a clean 250-mL glass impinger ("bubbler") and add 100 mL of Milli-Q water and 3 mL of 10% w/v $SnCl_2$ in 10% v/v HCl.
- Connect a drying column (soda lime trap) to the impinger gas exit and a gold trap downstream of the drying column.
- Add 100 pg of Hg from the working Hg standard solution. In the case of concentrations listed in the section 3.4, this means that 100.5 µL of working standard solution (Hg concentration of 0.9954 ng g^{-1}) needs to be pipetted directly to the impinger.
- As soon as the Hg standard is added, quickly close the impinger and connect it to a N_2 flow of 1 L min^{-1} for 10 minutes to purge the Hg^0 to the gold trap. Note: lower total volume of the solution in the impinger can be used together with a lower N_2 flow and shorter purging time since these parameters mostly depend on the shape, size, and design of the impinger.
- After 10 minutes, remove the Hg-loaded gold trap from the impinger and use it for the second step.

4.2 NTP oxidation of Hg^0 to Hg^{II} species

Similar setup is used as in Figure 2a of the manuscript's main body. Three different Hg^{II} species can be produced via NTP oxidation: HgO with O_2 reaction gas, $HgCl_2$ with Cl_2 reaction gas, and $HgBr_2$ with Br_2 reaction gas. Therefore, we can have three setups that marginally differ from each other.

- Assemble the following parts of the setup (similar as in Figure 2a in the manuscript's main body, going downstream in consecutive order): Hg-loaded gold trap, T-split for the introduction of reaction gas, plasma trap, and impinger with solution for reaction gas reduction. The reaction gas is obtained from i) O_2 gas cylinder for O_2 reaction gas and ii) electrolytic reaction for Cl_2 and Br_2 reaction gases (electrolysis described in section S4 of the Supplementary Information). The solution for reaction gas reduction is 50 mL of 10% w/v KOH for Cl_2 reaction gas and 50 mL of 10% w/v $Na_2S_2O_3$ for Br_2 reaction gas. In the case of O_2 reaction gas, the reduction of the reaction gas is not needed. The final flow of the gas mixture (He + reaction gas) should be 370 mL min^{-1}, where He represents >99% of the gas mixture and reaction gas represents <1% of the gas mixture.

- IMPORTANT NOTE: Cl_2 and Br_2 reaction gases are strong oxidants on their own (even without NTP). Therefore, it is important to release them into the system only when every part of the setup is gas-tight. Always connect the reaction gas the last (to avoid unnecessary reactions), after the whole setup is already on the flow of He gas.

- When the setup is assembled and under gas-tight He flow, connect the copper electrodes as shown on Figure 2a in the main text of the article, turn on the plasma driver and set it to these parameters: power applied to the electrodes of 180 µW, radiofrequency of 20 kHz, effective voltage of 345 V.

- After plasma is homogeneous, release the reaction gas flow and after approximately 10 seconds start heating the gold trap (e.g., using a heating coil) to 400 °C to release the Hg^0 from the trap.

- After heating is finished, wait for 60 s to ensure complete release of Hg^0 and then close the reaction gas flow and turn off plasma by turning off the plasma driver.

- After the reaction gas flow is closed and plasma driver is turned off, the setup can be safely disassembled. The produced Hg^{II} that is needed for further calibration is trapped on the plasma trap.

4.3 Thermal reduction of Hg^{II} species to Hg^0 and subsequent CV-AFS analysis

Similar setup is used as in Figure 2b of the manuscript's main body.

- Connect the plasma trap that is loaded with Hg^{II} species to a gold trap downstream.

- Connect both traps to the He flow of 370 mL min^{-1}.

- Heat the whole plasma trap (both KCl crystal part and Al_2O_3 catalyst) to >600 °C. The temperature should be achieved as fast as possible to avoid desorption of Hg^{II} and its re-deposition on cold spots of the system. For compete thermal reduction of Hg^{II} to Hg^0 (without Hg^{II} desorption), the plasma trap should be heated to >600 °C in under 20 s.

- After the heating is completed, wait for 60 seconds to ensure complete downstream transport of Hg^0 to the gold trap.

- The gold trap is now ready for the determination of mercury by double amalgamation CV-AFS measurement.[1]

S2 - Chemicals and instruments used in the validation work

Table S1. Chemicals used in the validation work.

Description of chemicals	Purity / type	Producer
65 % nitric acid (HNO_3)	For analysis	Supelco, Darmstadt, Germany
30 % hydrochloric acid (HCl)	Suprapur	Merck, Darmstadt, Germany
Tin(II) chloride dihydrate ($SnCl_2{\cdot}2H_2O$)	For analysis, max. 0.000001 % Hg	Merck, Darmstadt, Germany
Gold(III) chloride hydrate ($HAuCl_4{\cdot}xH_2O$)	99.995 % trace metal basis	Merck, Darmstadt, Germany
Potassium chloride (KCl) salt	Suprapur	Merck, Darmstadt, Germany
Mercury(II) oxide-yellow (HgO)	99.5 %	May & Baker, Essex, United Kingdom
Mercury(II) chloride ($HgCl_2$)	99.998 %	Merck, Darmstadt, Germany
Mercury(II) bromide ($HgBr_2$)	99.0 %	Merck, Darmstadt, Germany
NIST SRM 3133: Mercury (Hg) Standard Solution	9.954 mg g^{-1} ± 0.053 mg g^{-1}	NIST, Gaithersburg, MD, USA
^{196}Hg enriched elemental Hg	51.58 % ^{196}Hg	Isoflex, San Francisco, CA, USA
Type I purified water	Electrical resistivity 18.2 MΩ cm	Merck, Darmstadt, Germany

Table S2. Instruments used in the validation work.

Description of instruments	Model	Producer
High-voltage high-frequency power generator	Amazing PMV500	Information Unlimited, Amherst, NH, USA
High-voltage probe	Tektronix P6105A	Tektronix Inc., Beaverton, OR, USA
Electrical current monitor	Pearson 2877	Pearson Electronics Inc., Palo Alto, CA, USA
Oscilloscope	Picoscope 3204d	Pico Technology Ltd., Cambridgeshire, UK
High-purity germanium (HPGe) coaxial-type detector	Model 7229P	Canberra Industries Inc., Meriden, CT, USA
High-purity germanium (HPGe) well-type detector	Model GCW6023/S	Canberra Industries Inc., Meriden, CT, USA
Cold vapor atomic absorption spectrometer	Model Hg-201 Semi-Automated Mercury Analyzer	Sanso Seisakusho Co., Ltd., Tokyo, Japan
Quadrupole mass spectrometer (QMS)	Model QMS 700	Pfeiffer Vacuum Ltd., Asslar, Germany

S3 - Calculation of the decay time-corrected peak areas for samples and standards and the calculation of oxidation or thermal reduction efficiency

The peak area of the sample and standard activity had to be corrected for the decay since the start of irradiation. From the corrected peak areas for samples and standards, the oxidation or thermal reduction efficiencies were calculated as shown in the following paragraphs.

Eq. (1) was applied for calculation of both $A_{0,\,s}$ (sample peak area at reference time) and $A_{0,\,std}$ (standard peak area at reference time). If we insert the $A_{0,\,s}$ and $A_{0,\,std}$ values from Eq. (1) into the Eq. (2) (used for oxidation or thermal reduction efficiency calculation), we obtain Eq. (3).[2]

$$A_{0,i} = \frac{\mathrm{A}_i \cdot \frac{\ln 2}{t_{1/2}}}{e^{-\left(\frac{t_{passed} \cdot \ln 2}{t_{1/2}}\right)} \cdot \left[1 - e^{-\left(\frac{t_{measurement} \cdot \ln 2}{t_{1/2}}\right)}\right]} \tag{1}$$

where i is either sample (s) or standard (std).

$$\eta = \frac{A_{0,s}}{A_{0,std.}} \cdot 100 \cdot F_{dil} \cdot F_{rep} \tag{2}$$

$$\eta = \frac{A_s \cdot e^{-\left(\frac{t_{passed,std} \cdot \ln 2}{t_{1/2}}\right)} \cdot \left[1 - e^{-\left(\frac{t_{measurement,std} \cdot \ln 2}{t_{1/2}}\right)}\right]}{A_{std} \cdot e^{-\left(\frac{t_{passed,s} \cdot \ln 2}{t_{1/2}}\right)} \cdot \left[1 - e^{-\left(\frac{t_{measurement,s} \cdot \ln 2}{t_{1/2}}\right)}\right]} \cdot 100 \cdot F_{dil} \cdot F_{rep} \tag{3}$$

Where:

$A_{0,\,s}$ is the sample peak area at reference time t=0,

$A_{0,\,std}$ is the standard peak area at reference time t=0,

A_s is the sample peak area at the time of measurement,

A_{std} is the standard peak area at the time of measurement,

$t_{1/2}$ is the half-life of ^{197}Hg [s],

$t_{passed,\,s}$ is the time passed since reference time t=0 till the start of the sample measurement [s],

$t_{measurement,\,s}$ is the time passed during the sample measurement [s],

η is the oxidation or thermal reduction efficiency [%],

F_{dil} is the dilution factor,

F_{rep} is the repeatability factor (value of 1).

S4 - Electrolytic production of reaction gases Cl_2 and Br_2

Since Cl_2 and Br_2 are notoriously difficult to handle, they were produced in the laboratory just for the needs of our experiments. We used electrolysis of 1 M NaCl solution to produce Cl_2 and electrolysis of 1 M KBr solution to produce Br_2. 4.5 V battery was used as a power source for electrolysis. NaCl infused agar gel was used as a salt bridge to provide a closed electric circuit. The experimental setup for electrolytic production of Cl_2 and Br_2 is shown in Figure S1.

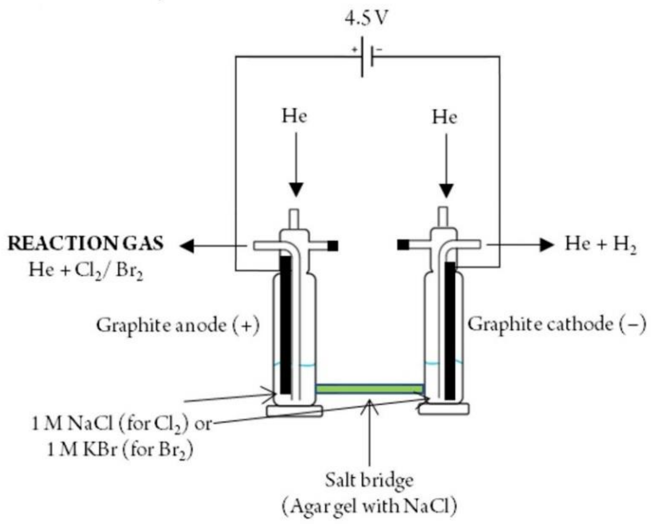

Figure S1. Experimental setup for electrolytic production of reaction gases Cl_2 and Br_2.

As Cl_2 and Br_2 are both water-soluble, the impinger solution containing the anode was always purged with He. This increased the amount of Cl_2 and Br_2 in the gas stream. As Br_2 immediately dissolves in the solution, it is not released into the gas stream until enough Br_2 is produced and its vapor pressure is sufficiently high.

S5 - All replicates for the Hg^{II} thermal reduction and production of Hg^{II} species by NTP.

Table S3. Hg^{II} to Hg^{o} thermal conversion, Hg^{II} loaded by spike, all replicates.

Catalyst used	Hg^{o}, thermal reduction efficiency [%]	Unconverted Hg^{II} [%]	Mass balance [%]
No catalyst	106	22.5	129
	69.1	28.6	97.7
Au-coated silica	34.5	65.5	100
	40.2	56.5	96.7
	39.0	61.0	100
Pt wire	80.2	1.92	82.1
	28.9	59.4	88.3
	26.5	64.8	91.3
	20.6	71.5	92.1
Quartz wool	98.0	5.33	103
	96.2	11.6	108
	64.7	27.8	92.5
Al_2O_3	106	<0.1	106
	102	<0.1	102
	97.4	<0.1	97.4
	98.6	<0.1	98.6
	99.1	<0.1	99.1

Table S4. Production of Hg^{II} species by NTP, all replicates.

Species used	Mass used [pg]	Unconverted Hg^{o} [%]	Hg^{II} on plasma trap, oxidation efficiency [%]
HgO	100	1.19	97.6
		2.22	101
		0.47	100
		2.26	102
		2.17	102
$HgCl_2$	250	2.30	93.2
		1.57	93.2
		1.60	99.7
		1.87	98.4
		3.71	99.3
$HgBr_2$	250	<0.1	79.3
		3.03	79.0
		1.91	68.8
		2.96	82.2

S6 - Calculation of the combined standard uncertainty for the proposed Hg^{II} species calibration

The uncertainty of the developed calibration was estimated according to the GUM and Eurachem guidelines.[3,4] The model used for uncertainty evaluation was shown in Eq. (3). To make the model clearer, we substituted the exponential terms:

$$EF_{p,std} = e^{-\left(\frac{t_{passed,std} \cdot \ln 2}{t_{1/2}}\right)} \tag{4}$$

$$EF_{p,s} = e^{-\left(\frac{t_{passed,s} \cdot \ln 2}{t_{1/2}}\right)} \tag{5}$$

$$EF_{m,std} = e^{-\left(\frac{t_{measurement,std} \cdot \ln 2}{t_{1/2}}\right)} \tag{6}$$

$$EF_{m,s} = e^{-\left(\frac{t_{measurement,s} \cdot \ln 2}{t_{1/2}}\right)} \tag{7}$$

This way, the model can be re-written as:

$$\eta = \frac{A_s \cdot EF_{p,std} \cdot [1 - EF_{m,std}]}{A_{std} \cdot EF_{p,s} \cdot [1 - EF_{m,s}]} \cdot 100 \cdot F_{dil} \cdot F_{rep} \tag{8}$$

The equations for the standard uncertainty of the exponential terms (e.g. $EF_{p,std}$) are as follows:

$$u(EF_{p,std}) = \frac{\partial EF_{p,std}}{\partial t_{1/2}} \cdot u\left(t_{1/2}\right) = EF_{p,std} \cdot \frac{ln2 \cdot t_{passed,std}}{\left(t_{1/2}\right)^2} \cdot u(t_{1/2}) \tag{9}$$

$$u(EF_{p,s}) = EF_{p,s} \cdot \frac{ln2 \cdot t_{passed,s}}{\left(t_{1/2}\right)^2} \cdot u(t_{1/2}) \tag{10}$$

$$u(EF_{m,std}) = EF_{m,std} \cdot \frac{ln2}{t_{1/2}} \cdot u(t_{measurement,std}) \tag{11}$$

$$u(EF_{m,s}) = EF_{m,s} \cdot \frac{ln2}{t_{1/2}} \cdot u(t_{measurement,s}) \tag{12}$$

Note that we assumed constant $t_{passed,\ std}$ and $t_{passed,\ s}$ to obtain Eq. (9) and Eq. (10) and constant $t_{1/2}$ to obtain Eq. (11) and Eq. (12). This assumption was made because in Eq. (9) and Eq. (10), the relative standard uncertainties of $t_{passed,\ std}$ and $t_{passed,\ s}$ were negligible in comparison to the relative standard uncertainty of $t_{1/2}$ (three orders of magnitude smaller relative standard uncertainties for $t_{passed,\ std}$ and $t_{passed,\ s}$ than for $t_{1/2}$). Similar assumption was made for Eq. (11) and Eq. (12); in this case, the relative standard uncertainty of $t_{1/2}$ was negligible in comparison to the relative standard uncertainty of $t_{measurement,\ std}$ and $t_{measurement,\ s}$.

The equation for obtaining standard uncertainties for A_s and A_{std} is calculated according to Poisson distribution with some adjustments due to background correction for the peak areas. The exact equation used for calculation is provided by the Genie 2000 gamma analysis software tool and is calculated automatically by the software, using Eq. (13).[5] Since ^{197}Hg has two doublet peaks in the activity measurement spectrum, the equation can be applied for both peaks, giving us two uncertainties u(A_1) and u(A_2). The standard uncertainty of the combined peaks (no overlapping of the peaks for our case) is calculated using Eq. (14).

$$u(A_i) = \sqrt{A_i + B_i \cdot \left(\frac{W_{p,i}}{W_{b,i}}\right)^2} \quad ; i = 1, 2 \tag{13}$$

$$u(A) = \frac{1}{\sqrt{u(A_1)^2 + u(A_2)^2}} \quad ; A = A_s \ or \ A_{std} \tag{14}$$

Where:
A_i is the sample peak area for the observed peak,
B_i is the background peak area for the observed peak,
$W_{p,i}$ is the spectral width of the sample peak region for the observed peak,
$W_{b,i}$ is the spectral width of the background peak region for the observed peak.

The last uncertainty components are present in F_{dil} and F_{rep}. u(F_{dil}) originates from two pipetting steps, one for the standard (5 mL pipette, u(V_{std})) and one for the sample (1 mL pipette, u(V_s)). Both u(V_{std}) and u(V_s) are calculated from uncertainty due to temperature influences (V_T) and uncertainty due to repeatability (V_{rep}); 10 replicate measurements) with Eq. (15). By combining both pipetting uncertainties, u(F_{dil}) is calculated with Eq. (16). F_{rep} value equals 1, but has to be included due to the components of the measurement procedure that influence the repeatability.

$$\frac{u(V_i)}{V_i} = \sqrt{\left(\frac{u(V_T)}{V_T}\right)^2 + \left(\frac{u(V_{i,rep})}{V_{i,rep}}\right)^2} \tag{15}$$

$$\frac{u(F_{dil})}{F_{dil}} = \sqrt{\sum_i \left(\frac{u(V_i)}{V_i}\right)^2} \tag{16}$$

where i is either sample (s) or standard (std).

u(F_{rep}) is the repeatability standard uncertainty and is calculated from the standard deviation of replicate oxidation efficiency measurements.

All listed uncertainty components are then used in calculation of combined standard measurement uncertainty by the law of uncertainty propagation. Combined standard measurement uncertainty is calculated as follows:

$$u_c(\eta) = \eta \cdot \sqrt{\begin{aligned} &\left(\frac{u(A_s)}{A_s}\right)^2 + \left(\frac{u(A_{std})}{A_{std}}\right)^2 + \left(\frac{u(EF_{p,s})}{EF_{p,s}}\right)^2 + \left(\frac{u(EF_{p,std})}{EF_{p,std}}\right)^2 \\ &+ \left(\frac{u(EF_{m,s})}{EF_{m,s}}\right)^2 + \left(\frac{u(EF_{m,std})}{EF_{m,std}}\right)^2 + \left(\frac{u(F_{dil})}{F_{dil}}\right)^2 + \left(\frac{u(F_{rep})}{F_{rep}}\right)^2 \end{aligned}} \tag{17}$$

The expanded standard uncertainty is obtained by multiplication of the combined standard measurement uncertainty with a coverage factor, k (k=2) with Eq. (18).

$$U = u_c(\eta) \cdot k \tag{18}$$

Table S5. Example: calculation of the combined standard measurement uncertainty for $HgCl_2$ species calibration

Parameter, c	Value	Assumed distribution	Standard uncertainty, u(c)	Comment
$t_{passed, s}$	714166 s	rectangular	/	Assumed negligible
$t_{passed, std}$	700920 s	rectangular	/	
$t_{measurement, s}$	150 s	rectangular	0.029 s	Standard uncertainty value used only for the calculation of $u(EF_i)$
$t_{measurement, std}$	150 s	rectangular	0.029 s	
$t_{1/2}$	230904 s	rectangular	404 s	
A_s	2020	Poisson	23.8	Parameters and their standard uncertainties that were used in calculation of combined standard uncertainty using the simplified model in Eq. (8)
A_{std}	2144	Poisson	20.1	
$EF_{p, std}$	0.122	rectangular	4.49E-04	
$EF_{p, s}$	0.117	rectangular	4.40E-04	
$EF_{m, std}$	0.9995	rectangular	6.04E-06	
$EF_{m, s}$	0.9995	rectangular	4.95E-05	
F_{dil}	1	Gaussian	0.004	
F_{rep}	1	Gaussian	0.034	

	Relative standard uncertainty u(c, r)	$u^2(c, r)$	Relative contribution	Combined standard uncertainty	Expanded standard uncertainty, k=2
A_s	1.18E-02	1.39E-04	9.78%	0.036	0.073
A_{std}	9.36E-03	8.76E-05	6.18%		
$EF_{p, std}$	3.68E-03	1.36E-05	0.96%		
$EF_{p, s}$	3.75E-03	1.41E-05	0.99%		
$EF_{m, std}$	6.04E-06	3.65E-11	0.00%		
$EF_{m, s}$	4.95E-05	2.45E-09	0.00%		
F_{dil}	4.48E-03	2.01E-05	1.42%		
F_{rep}	3.38E-02	1.14E-03	80.68%		

$\eta = 0.97 \pm 0.07$ (k=2)

Expanded standard uncertainty for the calibration of gaseous $HgCl_2$ by NTP oxidation is therefore 0.07 (k=2). Similar as for gaseous $HgCl_2$, we applied the above calculation for the uncertainty evaluation for gaseous HgO and gaseous $HgBr_2$. The expanded standard uncertainties amounted to 0.05 (k=2) for HgO and 0.09 (k=2) for $HgBr_2$.

References

(1) U.S. Environmental Protection Agency. EPA Method 1631, Revision E: Mercury in Water by Oxidation, Purge and Trap, and Cold Vapor Atomic Fluorescence. US Environmental Protection Agency 2002, p 38.

(2) Ribeiro Guevara, S.; Horvat, M. Stability and Behaviour of Low Level Spiked Inorganic Mercury in Natural Water Samples. *Anal. Methods* **2013**, *5* (8), 1996–2006. https://doi.org/10.1039/c3ay26496c.

(3) BIPM. *Evaluation of Measurement Data - Guide to the Expression of Uncertainty in Measurement*; 2008.

(4) Ellison, S. L. R.; Williams, A. Quantifying Uncertainty in Analytical Measurements, 3rd ed.; 2012.

(5) Zahn, G. S.; Genezini, F. A.; Moralles, M. Evaluation of Peak-Fitting Software for Gamma Spectrum Analysis. In *International Nuclear Atlantic Conference - INAC 2009*; Barros, R., Ed.; Rio de Janeiro, Brazil, 2011; pp 1083–1204.
